# A Public Warning

**Published:** 1912-11-02

**Authors:** Edward A. Attwood

**Affiliations:** Secretary. London Homœopathic Hospital.


### A Public Warning.

*To the Editor of* THE HOSPITAL.

SIR,—During the last few weeks I have received a number of letters and inquiries from our supporters in London and the provinces saying they have been called upon by young women—sometimes dressed in nurse's uniform—soliciting subscriptions on behalf of a "Cosmopolitan Homœopathic Institute."

To avoid misapprehension will you kindly allow me the courtesy of your columns to say that these collectors are in no way connected with the London Homœopathic Hospital, Great Ormond Street, and no portion of the funds collected ever reach our Treasurer, Lord Donoughmore, or, so far as I know, the treasurer of any recognised homœopathic hospital? When questioned, the collectors sometimes give a Bournemouth address as their headquarters, and on other occasions Croydon, Kennington, Hammersmith, etc., are given.

Neither the Bournemouth homœopathic physicians nor the physicians on the staff of the London Homœopathic Hospital or other recognised homœopathic physicians have any connection with this so-called Homœopathic Institute. —Yours truly, EDWARD A. ATTWOOD, Secretary.
London Homœopathic Hospital.

